# Supplementary material for: Identifying nonlinear dynamical systems via generative recurrent neural networks with applications to fMRI
Source: PLoS Comput Biol. 2019 Aug 21;15(8):e1007263. doi: 10.1371/journal.pcbi.1007263 (PMC6719895; doi:10.1371/journal.pcbi.1007263)
Supplement: S1 Text — (DOCX) [file pcbi.1007263.s001.docx]

**S1 Text. Model specification and inference.**

**PLRNN-BOLD-SSM model inference.** In the EM algorithm, we first aim to determine the posterior distribution $p(\mathbf{Z}|\mathbf{X})$ (E-Step), and – given this – then maximize the expectation of the joint ('complete data') log-likelihood $E_{q\mathbf{(Z}|\mathbf{X)}}[\log p(\mathbf{Z},\boldsymbol{X|\theta})]:=Q(\boldsymbol{\theta,Z})$ w.r.t. the parameters (M-Step). With the Gaussian noise assumptions (see eqns. 1-3, main manuscript), the expected joint log-likelihood is given by

1. $Q\left( \boldsymbol{\Theta,Z} \right)=-\frac{1}{2}{E_{q}[\left( \mathbf{z}_{1}-\boldsymbol{\mu}_{0}-\mathbf{C}\mathbf{s}_{1} \right)}^{T}\boldsymbol{\Sigma}^{-1}\left( \mathbf{z}_{1}-\boldsymbol{\mu}_{0}-\mathbf{C}\mathbf{s}_{1} \right)]$

$-\frac{1}{2}E_{q}[\sum_{t=2}^{T} \left( \mathbf{z}_{t}-\mathbf{A}\mathbf{z}_{t-1}-\mathbf{W}\varphi\left( \mathbf{z}_{t-1} \right)-\mathbf{h}-\mathbf{C}\mathbf{s}_{t} \right)^{T}\boldsymbol{\Sigma}^{-1}\left( \mathbf{z}_{t}-\mathbf{A}\mathbf{z}_{t-1}-\mathbf{W}\varphi\left( \mathbf{z}_{t-1} \right)-\mathbf{h}-\mathbf{C}\mathbf{s}_{t} \right)]$

$-\frac{1}{2}E_{q}[\sum_{t=1}^{T} \left( \mathbf{x}_{t}-\mathbf{B}\left( hrf\mathbf{*}\mathbf{z}_{\tau:t} \right)-\mathbf{Jr}_{t} \right)^{T}\boldsymbol{\Gamma}^{-1}\left( \mathbf{x}_{t}-\mathbf{B}\left( hrf\mathbf{*}\mathbf{z}_{\tau:t} \right)-\mathbf{J}\mathbf{r}_{t} \right)]-\frac{T}{2}(\log\left| \boldsymbol{\Sigma} \right|+\log\left| \boldsymbol{\Gamma} \right|)$,

where $\varphi\left( \mathbf{z}_{t} \right)\boldsymbol{:=}max(\mathbf{z}_{t},0)$ is an element-wise piecewise linear (ReLU) activation function.

The convolution with the hemodynamic response function (HRF) spells out as $hrf\mathbf{*}\mathbf{z}_{\tau:t}=\sum_{\tau=t-\Delta t}^{t} h_{t-\tau+1}\mathbf{z}_{\tau}$, where $h_{t-\tau+1}$ indexes the individual components of the HRF vector, and Δ*t*=0…*n*-1 depends on the temporal resolution of the time series, i.e. on the length n of the HRF vector.

**State estimation (E-Step).** We assume that $p(\mathbf{Z}|\mathbf{X})$, like a Gaussian, could be specified by its first two moments, i.e. the mean and the covariance, and that, as for a Gaussian, the MAP estimator is a reasonably good approximation to the mean. Thus we aim to maximize the log-joint distribution over $\mathbf{X}$ and $\mathbf{Z}$, $\log p(\mathbf{Z},\boldsymbol{X|\theta})$ (as defined by $Q_{\Omega}^{*}\left( \mathbf{Z} \right)$ in the main manuscript) w.r.t. $\mathbf{Z}$ [see also 17,88].

We have reformulated the optimization criterion in eq. 6 (main manuscript) in ‘big-matrix form’, defining the set of 'active' states (i.e., $\mathbf{z}_{t}>0$) through the binary vector $\mathbf{d}_{\Omega}:=I(\mathbf{z}>0)$. The matrix $\mathbf{H}\boldsymbol{\in}\mathbb{R}^{MTxMT}$ in eq. 6 is a convolution matrix which for *M*=1 contains the elements of the HRF in the following form

$$H=\left[\begin{matrix} \mathrm{h}_{\text{n}} & 0 & 0 & 0 & 0 & 0 & 0 & 0 & 0 \\ \mathrm{h}_{\text{n-1}} & \mathrm{h}_{\text{n}} & 0 & 0 & 0 & 0 & 0 & 0 & 0 \\ \mathrm{h}_{\text{n-2}} & \mathrm{h}_{\text{n-1}} & \mathrm{h}_{\text{n}} & 0 & 0 & 0 & 0 & 0 & 0 \\ \cdots& \mathrm{h}_{\text{n-2}} & \mathrm{h}_{\text{n-1}} & \mathrm{h}_{\text{n}} & 0 & 0 & 0 & 0 & 0 \\ \mathrm{h}_{1} & \cdots& \mathrm{h}_{\text{n-2}} & \mathrm{h}_{\text{n-1}} & \mathrm{h}_{\text{n}} & 0 & 0 & 0 & 0 \\ 0 & \mathrm{h}_{1} & \cdots& \mathrm{h}_{\text{n-2}} & \mathrm{h}_{\text{n-1}} & \mathrm{h}_{\text{n}} & 0 & 0 & 0 \\ 0 & 0 & \mathrm{h}_{1} & \cdots& \mathrm{h}_{\text{n-2}} & \mathrm{h}_{\text{n-1}} & \mathrm{h}_{\text{n}} & 0 & 0 \\ 0 & 0 & 0 & \mathrm{h}_{1} & \cdots& \mathrm{h}_{\text{n-2}} & \mathrm{h}_{\text{n-1}} & \mathrm{h}_{\text{n}} & 0 \\ 0 & 0 & 0 & 0 & \mathrm{h}_{1} & \cdots& \mathrm{h}_{\text{n-2}} & \mathrm{h}_{\text{n-1}} & \mathrm{h}_{\text{n}} \end{matrix}\right]$$

where $h_{i}$*, i=1…n,* denote the HRF vector elements.

For *M*>1, we would add additional columns by inserting *M*-1 zeros between each element of $\mathbf{H}$, and add additional rows by duplicating each row *M*-1 times, and shifting it by 1...*M*-1 positions, respectively.

Below we will also give the full structure of the block-banded matrices $\boldsymbol{\in}\mathbb{R}^{MTxMT}$ that occur in eq. 6, restated here for convenience:

$$Q_{\Omega}^{*}\left( \mathbf{Z} \right)=-\frac{1}{2}[\mathbf{z}^{\mathbf{T}}\left( \mathbf{U}_{0}+{\mathbf{D}_{\Omega}^{\mathbf{T}}\mathbf{U}}_{1}+{\mathbf{U}_{1}^{\mathbf{T}}\mathbf{D}}_{\Omega}+{\mathbf{D}_{\Omega}^{\mathbf{T}}\mathbf{U}}_{2}\mathbf{D}_{\Omega}+{\mathbf{H}^{\mathbf{T}}\mathbf{U}}_{3}\mathbf{H} \right)\mathbf{z-}\mathbf{z}^{\mathbf{T}}\left( \mathbf{v}_{0}+{\mathbf{d}_{\Omega}^{\mathbf{T}}\boldsymbol{\circ v}}_{1}+{\mathbf{H}^{\mathbf{T}}\mathbf{v}}_{2} \right)\mathbf{-}\left( \mathbf{v}_{0}+{\mathbf{d}_{\Omega}^{\mathbf{T}}\boldsymbol{\circ}\mathbf{v}}_{1}+{\mathbf{H}^{\mathbf{T}}\mathbf{v}}_{2} \right)^{\mathbf{T}}\mathbf{z}]+\mathrm{const}$$

Here, $\circ$ denotes the Hadamard product, all terms that do not depend on **z** are collected in $\mathrm{const}$, and the matrices and vectors are defined as follows:

$\mathbf{U}_{\boldsymbol{0}}\mathbf{=} \left[ \begin{matrix} \boldsymbol{\Sigma}^{-1}+\mathbf{A}^{T}\boldsymbol{\Sigma}^{-1}\mathbf{A} & -\mathbf{A}^{T}\boldsymbol{\Sigma}^{-1} & \boldsymbol{0} & \boldsymbol{\cdots} & \boldsymbol{0} \\ -\boldsymbol{\Sigma}^{-1}\mathbf{A} & \ddots& \ddots& \ddots& \boldsymbol{\vdots} \\ \boldsymbol{0} & \ddots& \ddots& \ddots& \boldsymbol{0} \\ \vdots& \ddots& \ddots& \boldsymbol{\Sigma}^{-1}+\mathbf{A}^{T}\boldsymbol{\Sigma}^{-1}\mathbf{A} & -\mathbf{A}^{T}\boldsymbol{\Sigma}^{-1} \\ \boldsymbol{0} & \boldsymbol{\cdots} & \boldsymbol{0} & -\boldsymbol{\Sigma}^{-1}\mathbf{A} & \boldsymbol{\Sigma}^{-1} \end{matrix} \right],$

$\mathbf{U}_{\boldsymbol{1}}\mathbf{=} \left[ \begin{matrix} \mathbf{W}^{T}\boldsymbol{\Sigma}^{-1}\mathbf{A} & -\mathbf{W}^{T}\boldsymbol{\Sigma}^{-1} & \boldsymbol{0} & \boldsymbol{\cdots} & \boldsymbol{0} \\ \boldsymbol{0} & \ddots& \ddots& \ddots& \boldsymbol{\vdots} \\ \vdots& \ddots& \mathbf{W}^{T}\boldsymbol{\Sigma}^{-1}\mathbf{A} & -\mathbf{W}^{T}\boldsymbol{\Sigma}^{-1} & \boldsymbol{0} \\ \vdots& \ddots& \boldsymbol{0} & \mathbf{W}^{T}\boldsymbol{\Sigma}^{-1}\mathbf{A} & -\mathbf{W}^{T}\boldsymbol{\Sigma}^{-1} \\ \boldsymbol{0} & \boldsymbol{0} & \boldsymbol{\cdots} & \boldsymbol{0} & \boldsymbol{0} \end{matrix} \right],$

$\mathbf{U}_{\boldsymbol{2}}\mathbf{=} \left[ \begin{matrix} \mathbf{W}^{T}\boldsymbol{\Sigma}^{-1}\mathbf{W} & \boldsymbol{0} & \boldsymbol{0} & \boldsymbol{\cdots} & \boldsymbol{0} \\ \boldsymbol{0} & \ddots& \ddots& \ddots& \boldsymbol{\vdots} \\ \vdots& \ddots& \mathbf{W}^{T}\boldsymbol{\Sigma}^{-1}\mathbf{W} & \ddots& \boldsymbol{0} \\ \vdots& \ddots& \boldsymbol{0} & \mathbf{W}^{T}\boldsymbol{\Sigma}^{-1}\mathbf{W} & \boldsymbol{0} \\ \boldsymbol{0} & \boldsymbol{0} & \boldsymbol{\cdots} & \boldsymbol{0} & \boldsymbol{0} \end{matrix} \right],$

$\mathbf{U}_{\boldsymbol{3}}\mathbf{=} \left[ \begin{matrix} \mathbf{B}^{T}\boldsymbol{\Gamma}^{-1}\mathbf{B} & \boldsymbol{0} & \boldsymbol{0} & \boldsymbol{\cdots} & \boldsymbol{0} \\ \boldsymbol{0} & \ddots& \ddots& \ddots& \boldsymbol{\vdots} \\ \vdots& \ddots& \ddots& \ddots& \boldsymbol{0} \\ \vdots& \ddots& \boldsymbol{0} & \mathbf{B}^{T}\boldsymbol{\Gamma}^{-1}\mathbf{B} & \boldsymbol{0} \\ \boldsymbol{0} & \boldsymbol{0} & \boldsymbol{\cdots} & \boldsymbol{0} & \mathbf{B}^{T}\boldsymbol{\Gamma}^{-1}\mathbf{B} \end{matrix} \right],$

$\mathbf{v}_{\boldsymbol{0}}\mathbf{=} \left[ \begin{matrix} \boldsymbol{\Sigma}^{-1}\mathbf{C}\mathbf{s}_{1}-\mathbf{A}^{T}\boldsymbol{\Sigma}^{-1}\left( \mathbf{C}\mathbf{s}_{2}\boldsymbol{+}\boldsymbol{\theta} \right)\boldsymbol{+}\boldsymbol{\Sigma}^{-1}\boldsymbol{\mu}_{0} \\ \vdots\\ \boldsymbol{\Sigma}^{-1}\left( \mathbf{C}\mathbf{s}_{t}\boldsymbol{+}\boldsymbol{\theta} \right)-\mathbf{A}^{T}\boldsymbol{\Sigma}^{-1}\left( \mathbf{C}\mathbf{s}_{t+1}\boldsymbol{+}\boldsymbol{\theta} \right) \\ \vdots\\ \boldsymbol{\Sigma}^{-1}\left( \mathbf{C}\mathbf{s}_{T}\boldsymbol{+}\boldsymbol{\theta} \right) \end{matrix} \right]$,

$\mathbf{v}_{\boldsymbol{1}}\mathbf{=} \left[ \begin{matrix} -\mathbf{W}^{T}\boldsymbol{\Sigma}^{-1}\left( \mathbf{C}\mathbf{s}_{2}\boldsymbol{+}\boldsymbol{\theta} \right) \\ -\mathbf{W}^{T}\boldsymbol{\Sigma}^{-1}\left( \mathbf{C}\mathbf{s}_{t+1}\boldsymbol{+}\boldsymbol{\theta} \right) \\ \vdots\\ -\mathbf{W}^{T}\boldsymbol{\Sigma}^{-1}\left( \mathbf{C}\mathbf{s}_{T}\boldsymbol{+}\boldsymbol{\theta} \right) \\ \boldsymbol{0} \end{matrix} \right]$,

$\mathbf{v}_{\mathbf{2}}\mathbf{=} \left[ \begin{matrix} \mathbf{B}^{T}\boldsymbol{\Gamma}^{-1}\mathbf{x}_{1}\boldsymbol{-}\mathbf{B}^{T}\boldsymbol{\Gamma}^{-1}\mathbf{Jr}_{1} \\ \vdots\\ \mathbf{B}^{T}\boldsymbol{\Gamma}^{-1}\mathbf{x}_{t}\boldsymbol{-}\mathbf{B}^{T}\boldsymbol{\Gamma}^{-1}\mathbf{Jr}_{t} \\ \vdots\\ \mathbf{B}^{T}\boldsymbol{\Gamma}^{-1}\mathbf{x}_{T}\boldsymbol{-}\mathbf{B}^{T}\boldsymbol{\Gamma}^{-1}\mathbf{Jr}_{T} \end{matrix} \right]$.

**Parameter estimation** (**M-Step**). In the M-Step, we maximize $Q(\boldsymbol{\theta,Z})$ given the state expectations returned by the E-Step w.r.t. to the parameters, which can be done analytically. The closed-form solution for the observation model parameters was given in the main manuscript; here we add the solutions for the latent model parameters. Specifically, we solve for $\mathbf{A}$, $\mathbf{W}$, $\mathbf{h}$, and $\mathbf{C}$ simultaneously by arranging these parameters horizontally within a matrix $\mathbf{L:=}\left[ \mathbf{A W h C} \right]\boldsymbol{\in}\mathbb{R}^{Mx(2M+1+K)}$, and defining the vector of predictor variables as $\mathbf{o}_{t}\boldsymbol{:=}\left[ \mathbf{z}_{t-1}\boldsymbol{,}\varphi\left( \mathbf{z}_{t-1} \right)\boldsymbol{,}1\boldsymbol{,}\mathbf{s}_{t} \right]\boldsymbol{\in}\mathbb{R}^{(2M+1+K)x1}$.

Since matrices $\mathbf{A}$ and $\mathbf{W}$ are not full (but contain to-be-estimated parameters only along the diagonal or off the diagonal, respectively), we solve for each row j of $\mathbf{L}$ in turn as

1. $\mathbf{L}_{j,k(j)}=\left( \sum_{t=2}^{T} E\left[ z_{j,t}\mathbf{o}_{k\left( j \right),t}^{T} \right] \right)\left( \sum_{t=2}^{T} E\left[ {\mathbf{o}_{k\left( j \right),t}\mathbf{o}}_{k\left( j \right),t}^{T} \right] \right)^{-1}$

where *k(j)* is a set of row indices which pick out those rows in $\mathbf{o}_{t}$ that correspond to those parameters actually defined, i.e. which squeeze out all zeros from the *j*’th row of $\mathbf{L}$, and accordingly from the respective rows/columns on the r.h.s. of eq. 2.

Let us define the following expectation sums:

$\mathbf{E}_{1}:=\sum_{t=2}^{T} E[\varphi\left( \mathbf{z}_{t-1} \right)\varphi\left( \mathbf{z}_{t-1} \right)^{T}]$, $\mathbf{E}_{2}:=\sum_{t=2}^{T} E[\mathbf{z}_{t}{\mathbf{z}_{t-1}}^{T}]$, $\mathbf{E}_{3}:=\sum_{t=2}^{T} E[\mathbf{z}_{t-1}{\mathbf{z}_{t-1}}^{T}]$,

$\mathbf{E}_{4}:=\sum_{t=2}^{T} E[\varphi\left( \mathbf{z}_{t-1} \right){\mathbf{z}_{t-1}}^{T}]$, $\mathbf{E}_{5}:=\sum_{t=2}^{T} E[\mathbf{z}_{t}{\varphi\left( \mathbf{z}_{t-1} \right)}^{T}]$, $\mathbf{F}_{3}:=\sum_{t=2}^{T} \mathbf{s}_{t}E[{\mathbf{z}_{t-1}}^{T}],$

$\mathbf{F}_{4}:=\sum_{t=2}^{T} \mathbf{s}_{t}E\left[ \varphi\left( \mathbf{z}_{t-1} \right)^{T} \right],$ $\mathbf{F}_{5}:=\sum_{t=2}^{T} E\left[ \mathbf{z}_{t} \right]{\mathbf{s}_{t}}^{T}$, $\mathbf{F}_{6}:=\sum_{t=2}^{T} \mathbf{s}_{t}{\mathbf{s}_{t}}^{T}$,

$\mathbf{G}_{1\Delta}:=\sum_{t=1+\Delta}^{T-1+\Delta} E\left[ \mathbf{z}_{t} \right]$, $\mathbf{G}_{2}:=\sum_{t=2}^{T} {\mathbf{s}_{t}}^{T},$ $\mathbf{G}_{3}:=\sum_{t=2}^{T} E\left[ \varphi\left( \mathbf{z}_{t-1} \right) \right].$

With this, the vector and matrix in eq. 2 can be written as

1. $\sum_{t=2}^{T} E\left[ z_{j,t}\mathbf{o}_{t}^{T} \right]=\left[ \mathbf{E}_{2,j} \mathbf{E}_{5,j} \mathbf{G}_{11,j} \mathbf{F}_{5,j} \right],$

and

1. $\sum_{t=2}^{T} E\left[ {\mathbf{o}_{t}\mathbf{o}}_{t}^{T} \right]=\left[ \begin{matrix} \mathbf{E}_{3} & \mathbf{E}_{4}^{T} & \mathbf{G}_{10} & \mathbf{F}_{3}^{T} \\ \mathbf{E}_{4} & \mathbf{E}_{1} & \mathbf{G}_{3} & \mathbf{F}_{4}^{T} \\ \mathbf{G}_{10}^{T} & \mathbf{G}_{3}^{T} & T-1 & \mathbf{G}_{2} \\ \mathbf{F}_{3} & \mathbf{F}_{4} & \mathbf{G}_{2}^{T} & \mathbf{F}_{6} \end{matrix} \right]$,

from which we select the columns *k(j)* from eq. 3, and the rows and columns *k(j)* out of eq. 4 to produce the solution for the *j-*th row in eq. 2.

Finally, the estimate for the initial condition is given by

$$\boldsymbol{\mu}_{0}=E\left[ \mathbf{z}_{1} \right]-\mathbf{C}\mathbf{s}_{1}.$$
