# Supplementary figures and images for: Identifying nonlinear dynamical systems via generative recurrent neural networks with applications to fMRI
Source: PLoS Comput Biol. 2019 Aug 21;15(8):e1007263. doi: 10.1371/journal.pcbi.1007263 (PMC6719895; doi:10.1371/journal.pcbi.1007263)

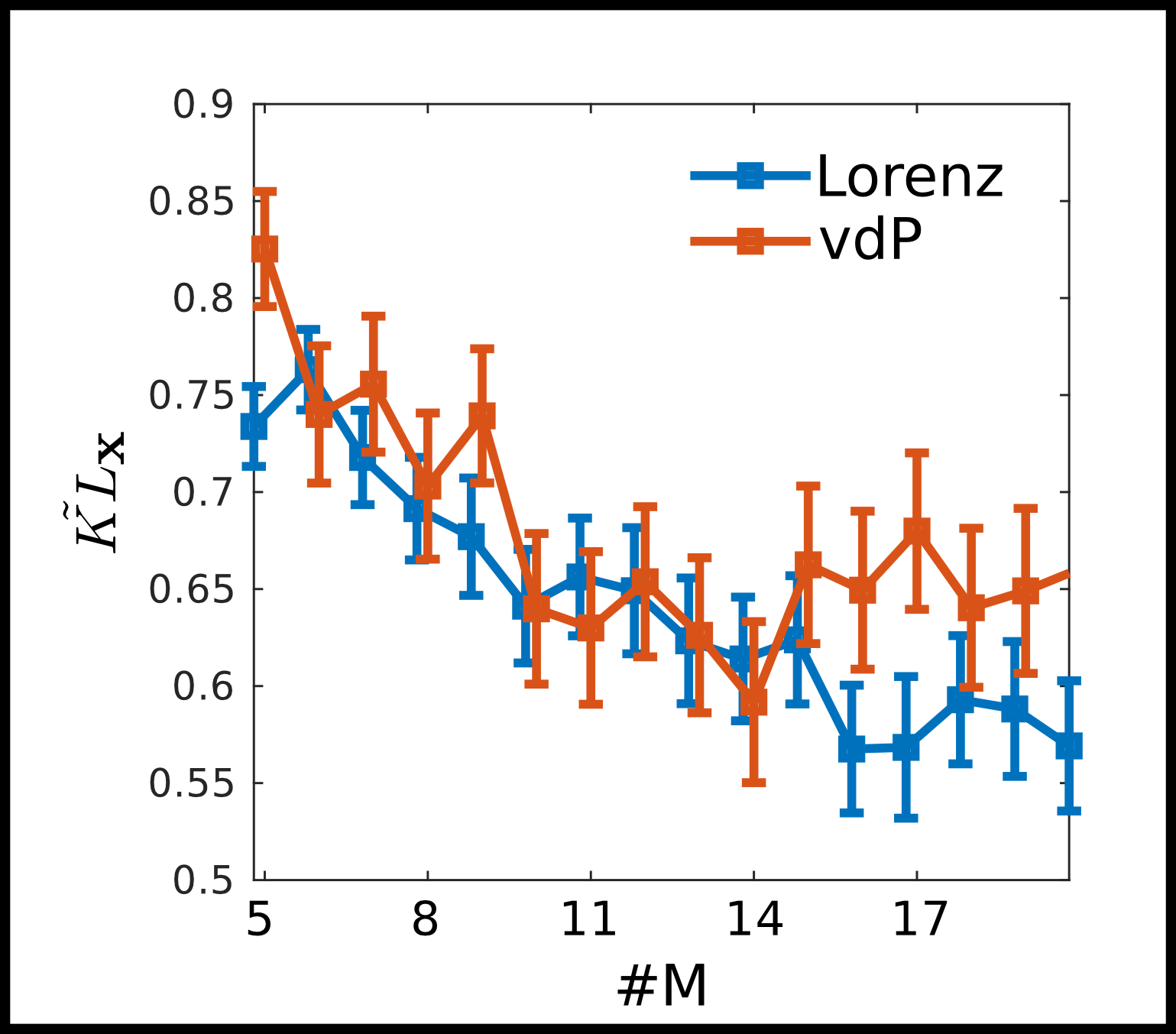

Supplement: S1 Fig — M = 14 seems to be about optimal for vdP, while M≈16 may be about optimal for the Lorenz system. (TIF) [file pcbi.1007263.s002.tif]

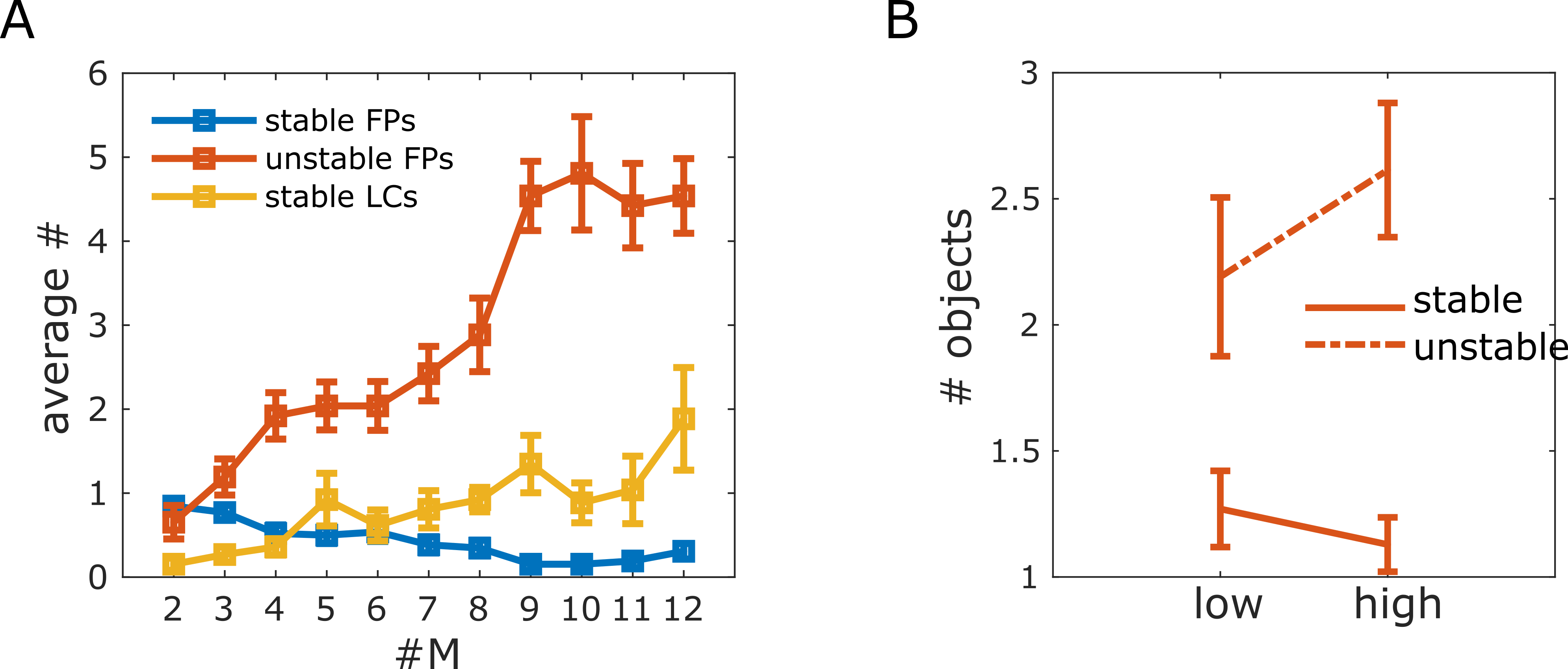

Supplement: S2 Fig — A. Number of stable (fixed points [FPs], limit cycles [LCs]) and unstable (fixed points) dynamical objects as a function of latent space dimensionality M. B. Same as Fig 10B for data pooled across M = 2…10 (repeated measures ANOVA for ‘performance x stability’ interaction: F(1,24) = 2.49, p = .128). (TIF) [file pcbi.1007263.s003.tif]

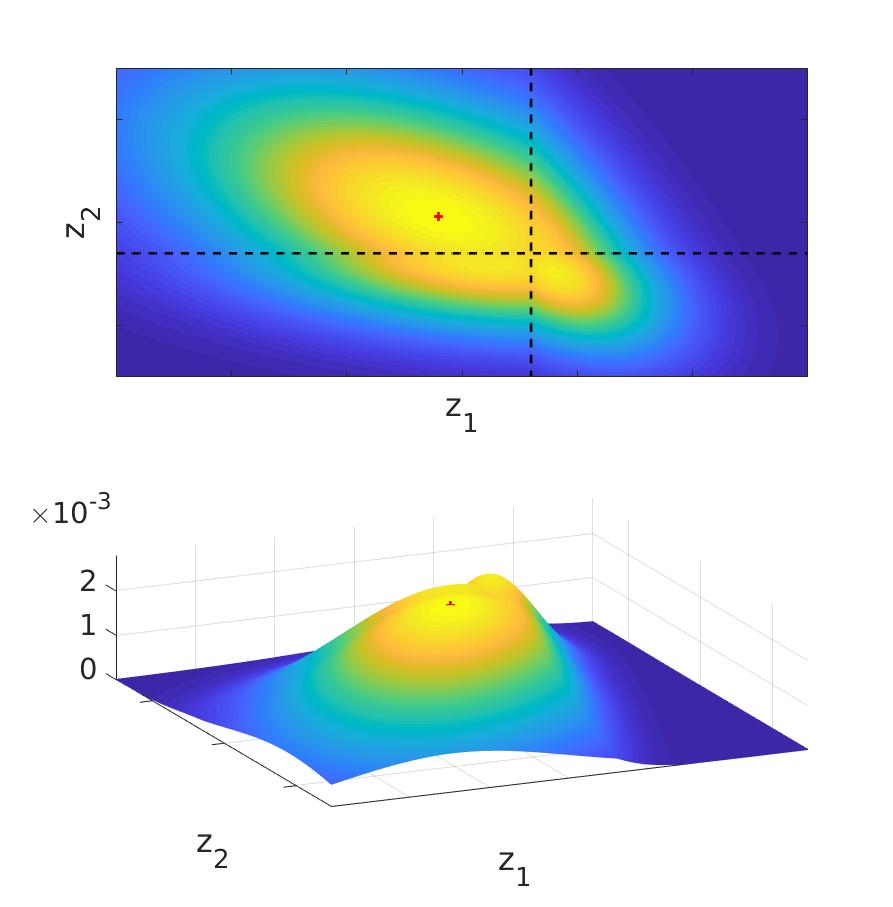

Supplement: S3 Fig — Illustration of the model’s likelihood landscape as a function of a single latent state across two consecutive time steps, z1 and z2. The joint likelihood p(X,Z) consists of piecewise Gaussians which cut off at the zeros of the states; often they will cluster near the origin and give rise to a strongly elevated plateau of high-likelihood solutions, close to one full Gaussian. Red cross indicates MAP estimate. (TIF) [file pcbi.1007263.s004.tif]

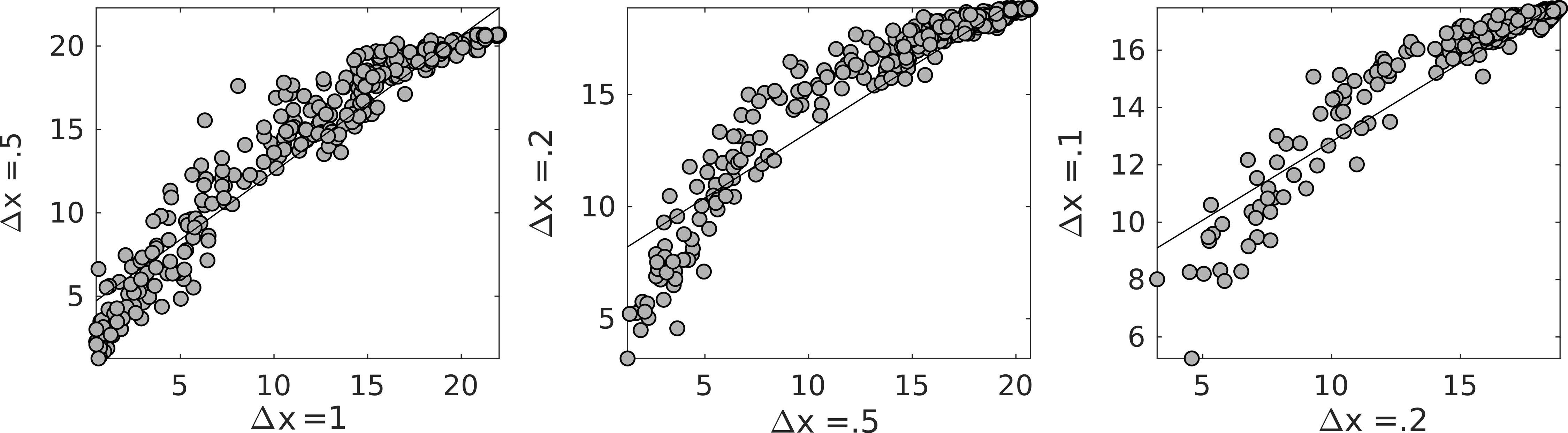

Supplement: S4 Fig — A. KLx for bin size Δx = 1 (x-axis) against bin size Δx = .5 (y-axis). B. Same as A for bin size Δx = .5 (x-axis) against Δx = .2 (y-axis). C. Same as A. for bin size Δx = .2 (x-axis) against Δx = .1 (y-axis). Measures at different bin sizes are nearly monotonically related such that rank information on the quality of DS retrieval is conserved. However, the KLx spread is largest for Δx = 1 such that qualitative differences in DS retrieval are differentiated more easily for this bin size, and hence this bin size was chosen for the evaluation in the main manuscript. (TIF) [file pcbi.1007263.s005.tif]
